# Supplementary material for: Multiple stages of evolutionary change in anthrax toxin receptor expression in humans
Source: Nat Commun. 2021 Nov 15;12:6590. doi: 10.1038/s41467-021-26854-z (PMC8592990; doi:10.1038/s41467-021-26854-z)
Supplement: Supplementary file 1 — Supplementary Information [file 41467_2021_26854_MOESM1_ESM.pdf]

Supplementary Information for: **Multiple stages of evolutionary change in anthrax toxin receptor expression in humans**

Lauren A. Choate[1,2], Gilad Barshad[1], Pierce W. McMahon[1], Iskander Said[2], Edward J. Rice[1], Paul R. Munn[1], James J. Lewis[1,*], and Charles G. Danko[1,3,*]

[1] Baker Institute for Animal Health, College of Veterinary Medicine, Cornell University, Ithaca, NY 14853.

[2] Department of Molecular Biology and Genetics, Cornell University, Ithaca, NY 14853.

[3] Department of Biomedical Sciences, College of Veterinary Medicine, Cornell University, Ithaca, NY 14853.

Address correspondence to:
Charles G. Danko, Ph.D.
Baker Institute for Animal Health
Cornell University
Hungerford Hill Rd.
Ithaca, NY 14853
Phone: (607) 256-5620
E-mail: dankoc@gmail.com

James J. Lewis
E-mail: jjl336@cornell.edu

Supplementary Figures 1-20. Supplementary Tables 1-2.

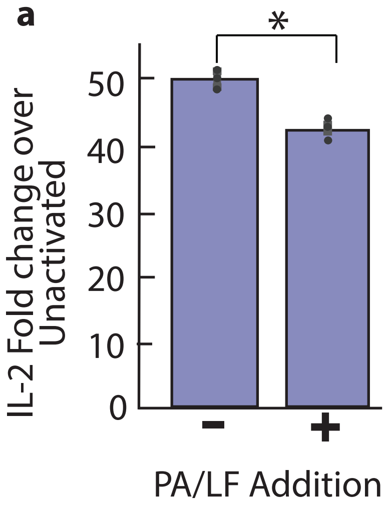

Supplementary Figure 1. Effect of anthrax toxin treatment on T-cell activation. a) CD4+ T-cells produce significantly less interleukin-2 (IL-2) upon activation with PMA and ionomycin when treated with anthrax toxins, protective antigen (PA) and lethal factor (LF), as measured by enzyme-linked immunoassay (ELISA) ($p<0.009$, one-sided t-test). IL-2 production was measured as fold change over samples that were not activated. Data are presented as mean values +/- SE ($n=3$ for each condition). Source data are provided as a Source Data file.

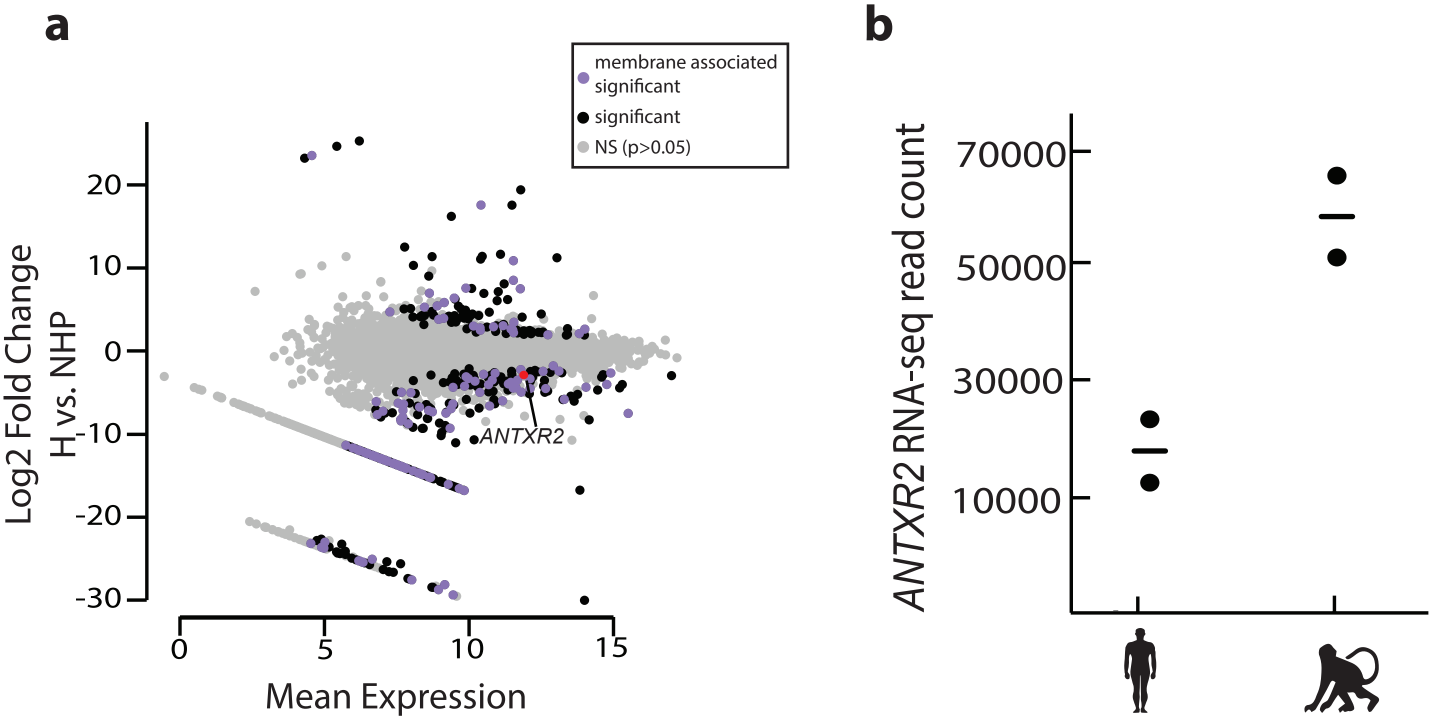

Supplementary Figure 2. Differential RNA expression between humans (H) and non-human primates (NHP). a) RNA-seq comparison of protein-coding genes between humans and rhesus macaque in CD4+ T-cells. The GO term ‘integral component of the membrane’ is enriched. *ANTXR2* is highlighted in red. Differential expression is based on DESeq2, $p<0.0001$, multiple testing correction made. b) *ANTXR2* RNA-seq in CD4+ T-cells is consistent with the relative levels of *ANTXR2* transcription based on PRO-seq with higher expression in rhesus macaque. Source data are provided as a Source Data file.

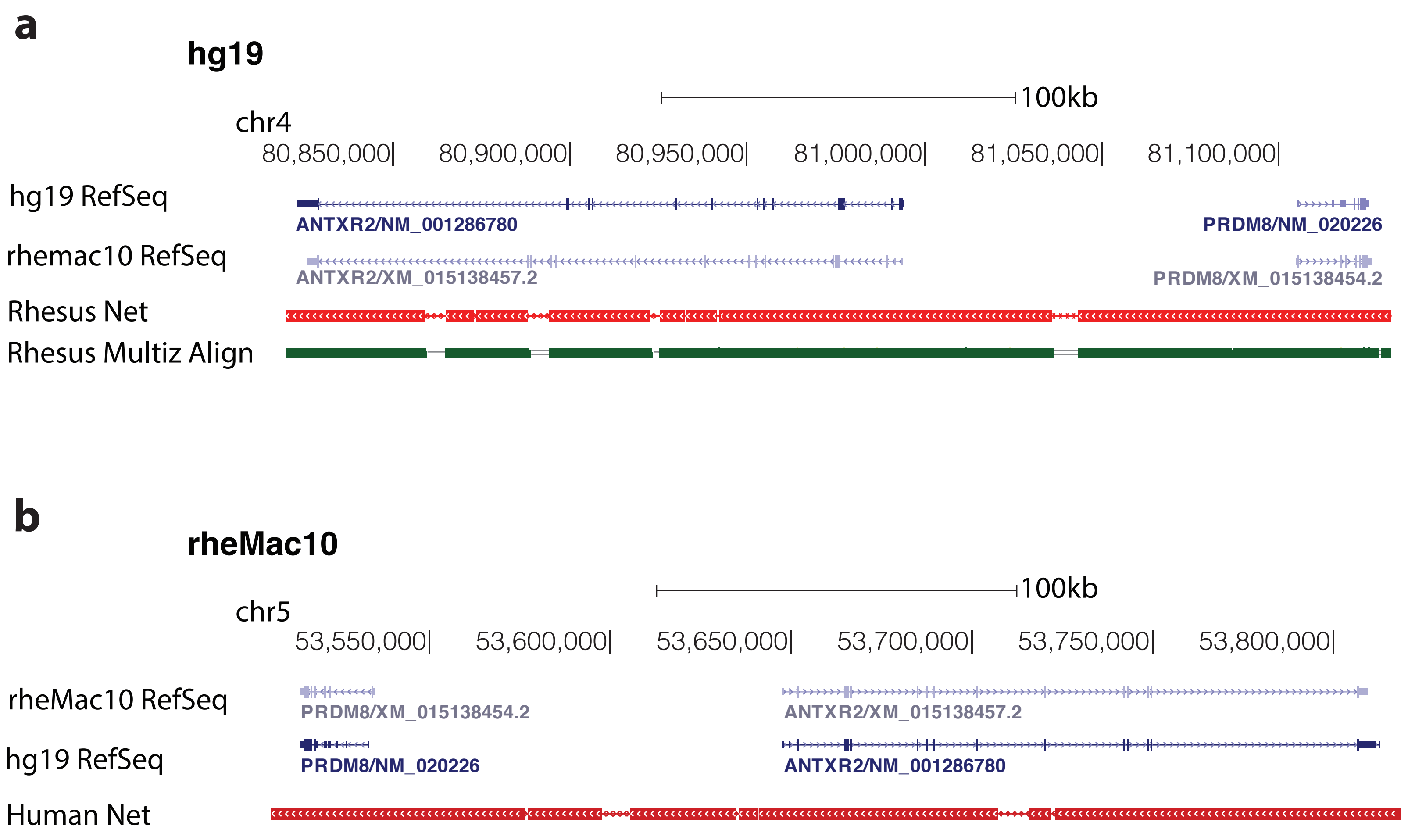

Supplementary Figure 3. Comparison of human and rhesus macaque *ANTXR2* loci. a) Human and rhesus macaque genes aligned to hg19. Rhesus net synteny is pictured in red. b) Human and rhesus macaque genes aligned to rheMac10. Human net synteny is pictured in red.

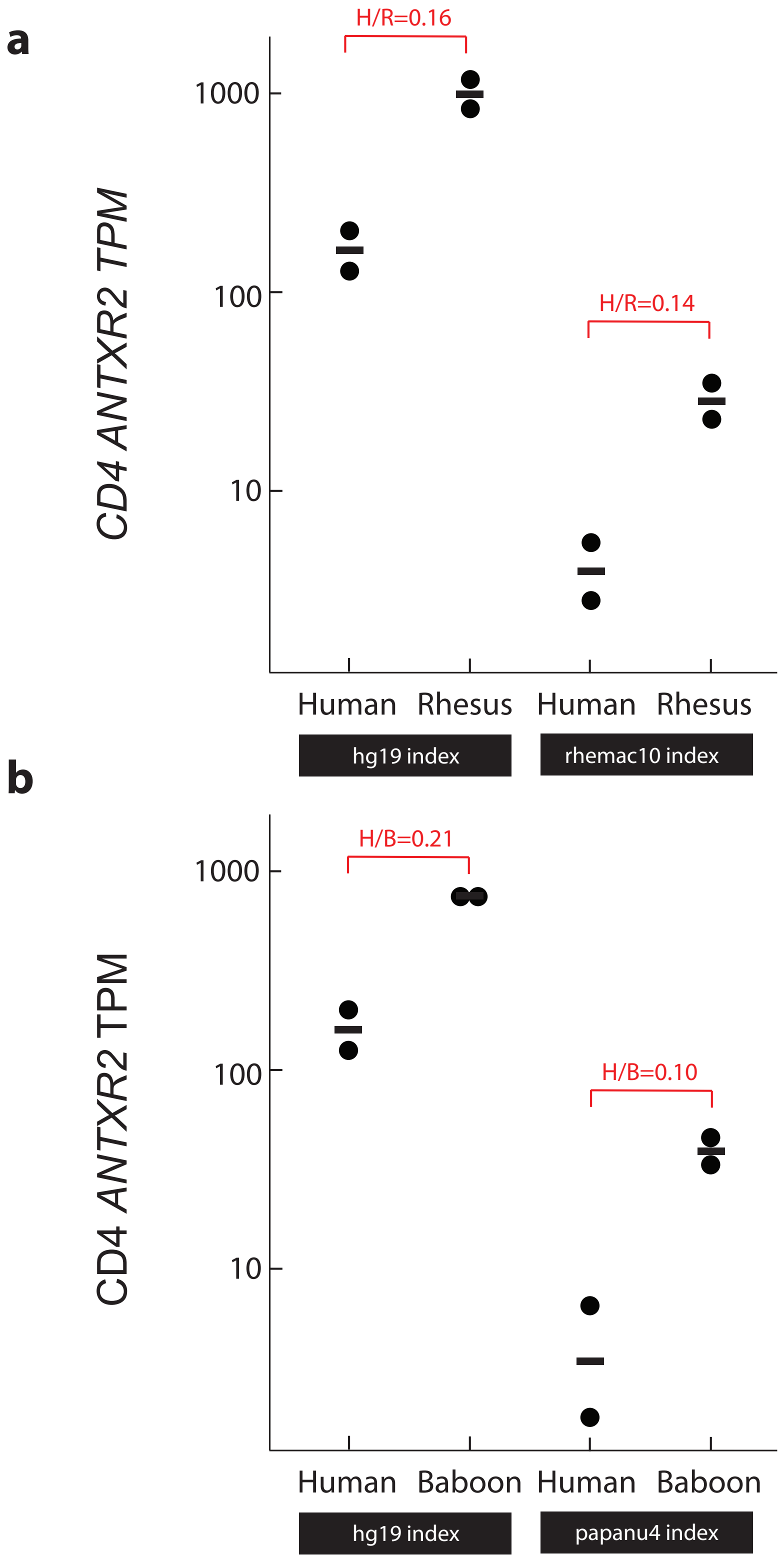

Supplementary Figure 4. Reciprocal analysis of Salmon between genomes. a) Comparison of Salmon estimates of *ANTXR2* expression using either the human or rhesus macaque transcriptome. Each species was mapped to both hg19 and rheMac10. There are similar ratios between human and rhesus (H/R) in the reciprocal analyses (H/R=0.16 mapped to hg19, H/R=0.14 mapped to rheMac10). b) Comparison of Salmon estimates using either the human or baboon transcriptome. Each species used transcript annotations from both hg19 and pananu4 in each comparison. There are similar ratios between the reciprocal analyses (H/B=0.21 mapped to hg19, H/B=0.10 mapped to papanu4) that suggest mapping to hg19 may be a conservative estimate of the true difference in expression. Source data are provided as a Source Data file.

a

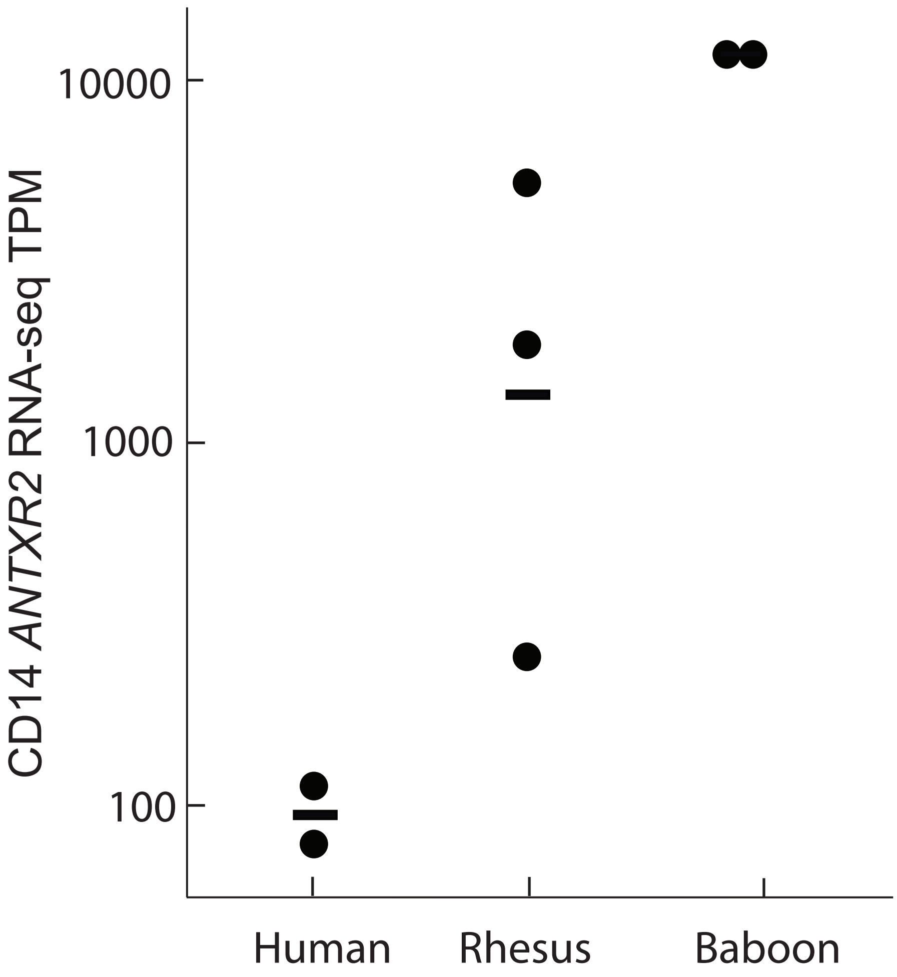

Supplementary Figure 5. *ANTXR2* RNA expression in CD14+ monocytes. a) RNA-seq TPM in *ANTXR2* calculated using Salmon in CD14+ monocytes for human, rhesus macaque, and baboon. Human expression of *ANTXR2* is significantly less than non-human primate (rhesus macaque and baboon grouped) (p=0.03, one-sided t-test, human n=2, rhesus n=3, baboon n=2). Source data are provided as a Source Data file.

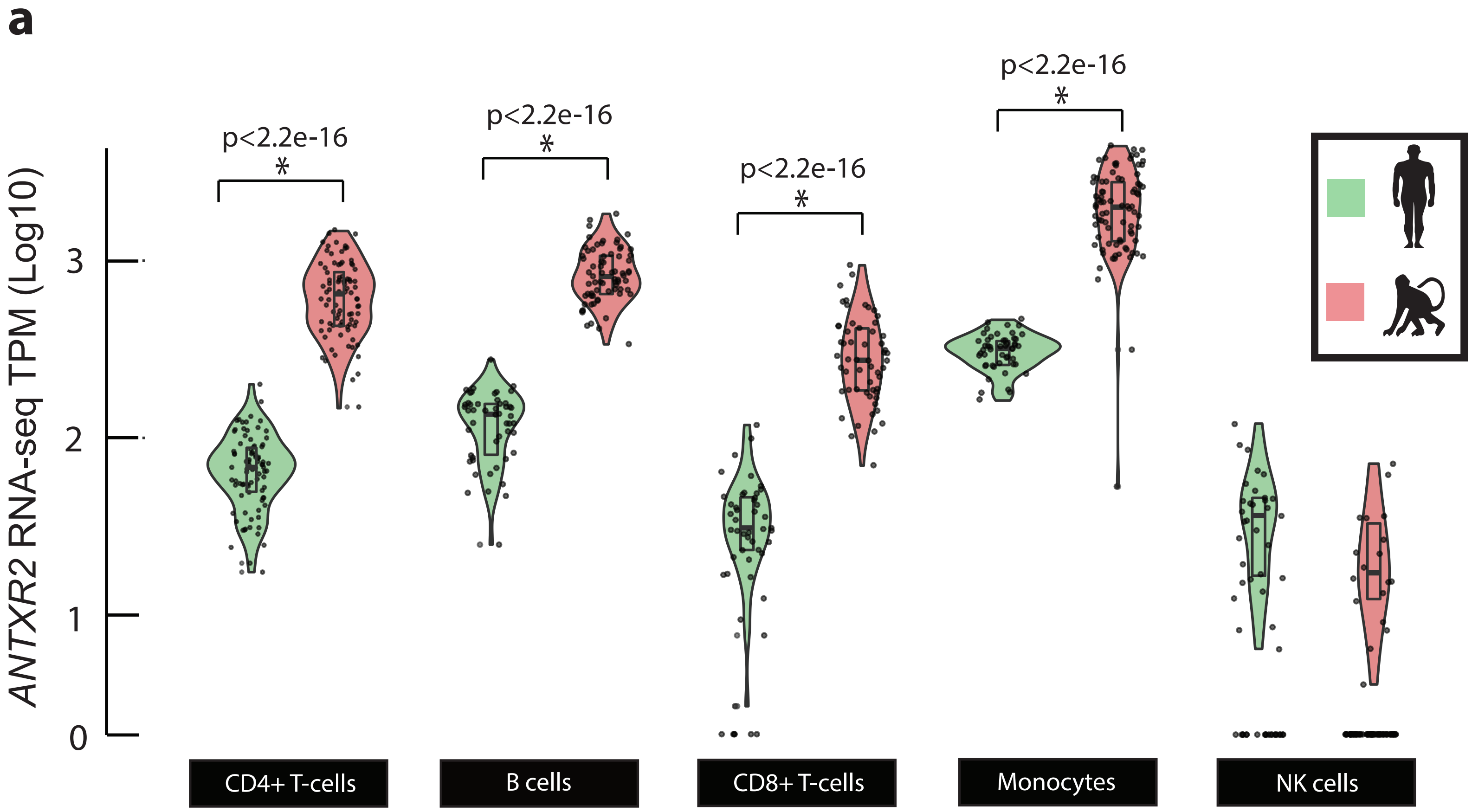

Supplementary Figure 6. Comparison of *ANTXR2* RNA expression in immune cells between rhesus macaque and human. a) RNA-seq TPM in *ANTXR2* calculated using Salmon in CD4+ T-cells, B cells, CD8+ T-cells, monocytes, and natural killer (NK) cells for rhesus macaque and human. Based on a one-sided Wilcoxon rank sum test, *ANTXR2* expression is significantly decreased in human compared to rhesus macaque in CD4+ T-cells, B cells, CD8+ T-cells, and monocytes (p=2.2x10^-16). Source data are provided as a Source Data file.

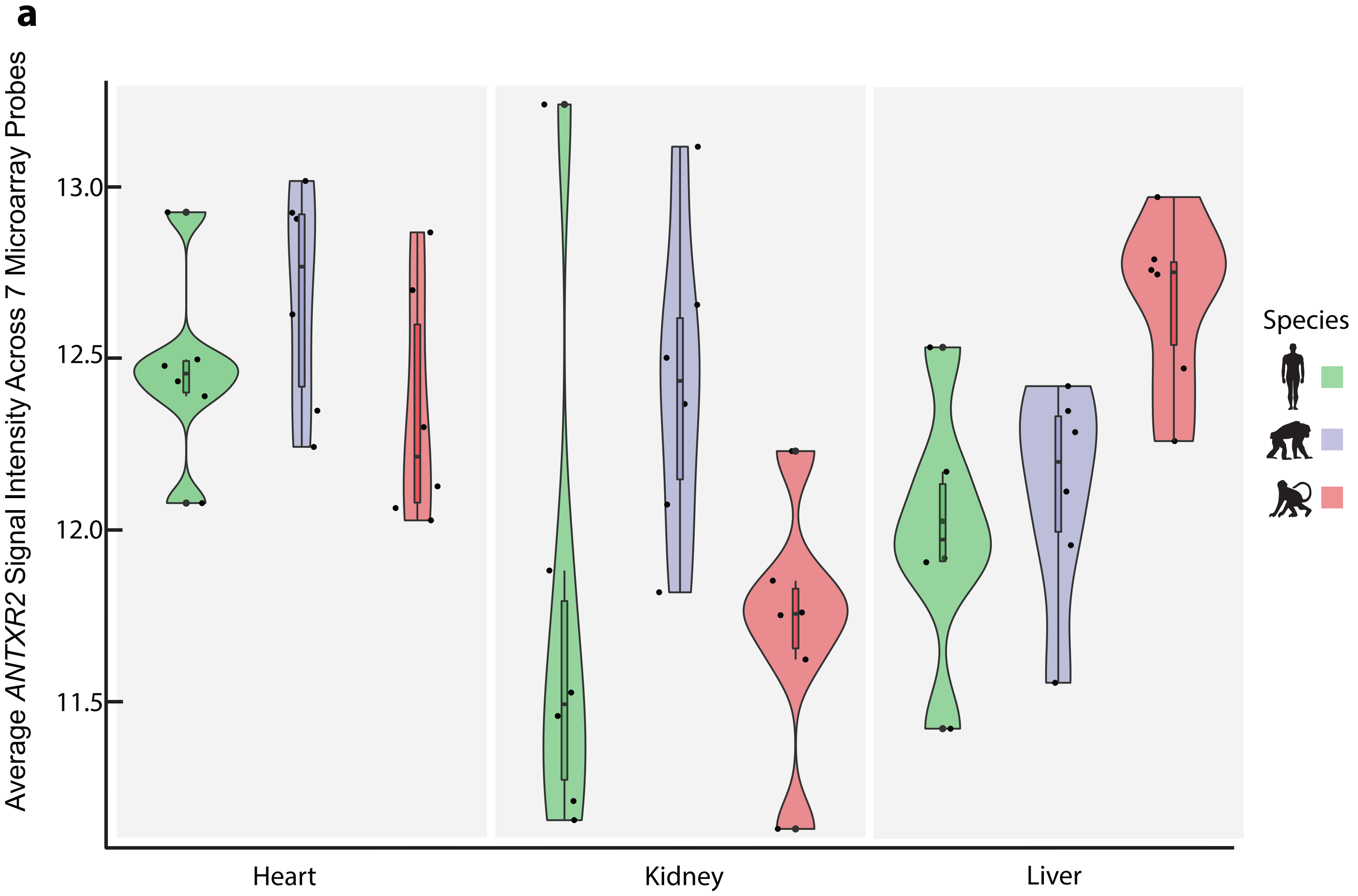

Supplementary Figure 7. Comparison of human, rhesus macaque, and chimpanzee *ANTXR2* expression across tissues. a) Microarray data in human, chimpanzee, and rhesus macaque heart, liver, and kidney shows no significant change across humans and non-human primates for *ANTXR2* according to the metric used by Blekhman et al. 2009. Source data are provided as a Source Data file.

a

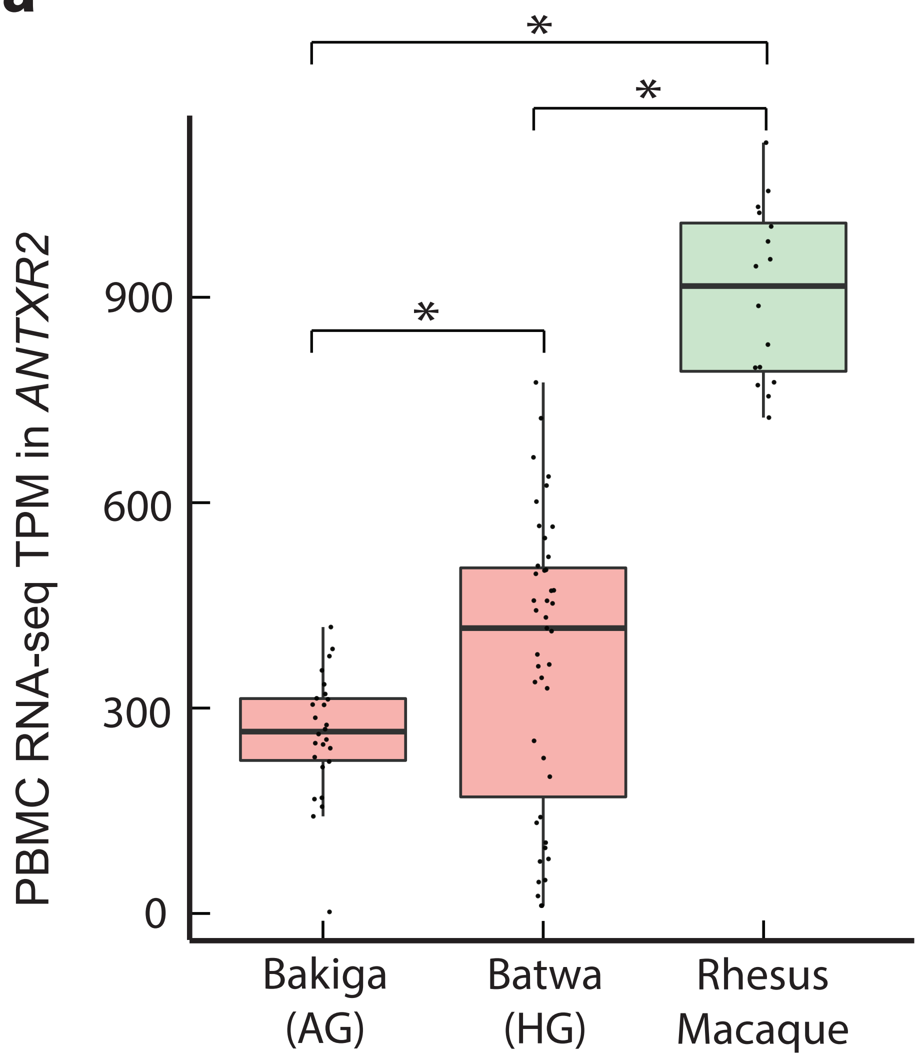

Supplementary Figure 8. Comparison of ANTXR2 RNA expression in PBMCs. a) RNA-seq in peripheral blood mononuclear cells (PBMCs) from Bakiga (agricultural (AG)) and Batwa (hunter-gatherer (HG)) individuals compared to rhesus macaque PBMCs. *ANTXR2* expression is significantly different for each comparison based on a one-sided Wilcoxon rank sum test (Bakiga vs. Batwa p=0.01; Bakiga vs. rhesus macaque p=1.2x10^-11; Batwa vs. rhesus macaque p=1.3x10^-13). n=44 for Batwa samples, n=25 for Bakiga samples, and n=16 for rhesus macaque samples. The bold center line denotes the median value (50th percentile) and the boxes contain the 25th to 75th percentile of the data. The whiskers mark 1.5 times the interquartile range (IQR). Source data are provided as a Source Data file.

a

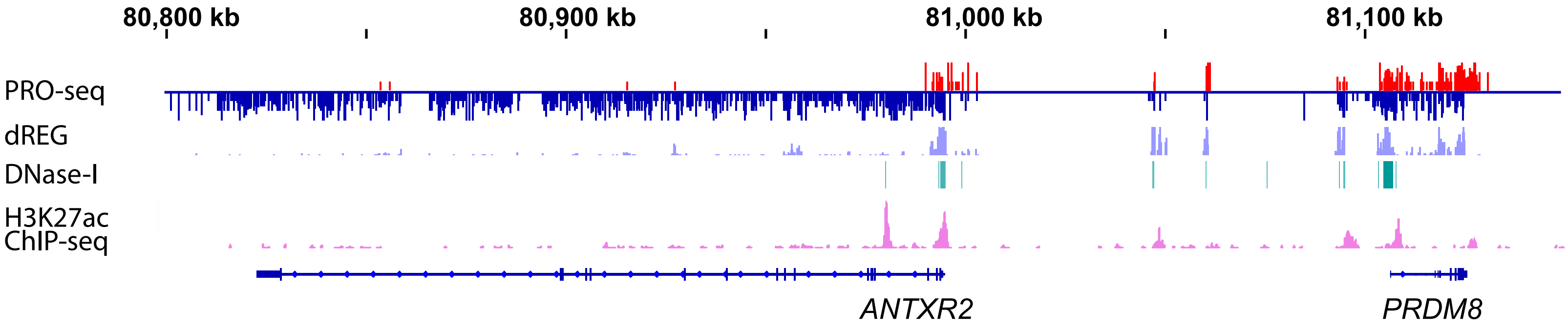

Supplementary Figure 9. H3K27ac and DNase-I-seq at *ANTXR2*. a) PRO-seq, dREG signal, DNase-I-seq peaks, and H3K27ac ChIP-seq at the *ANTXR2* locus from human CD4+ T-cells.

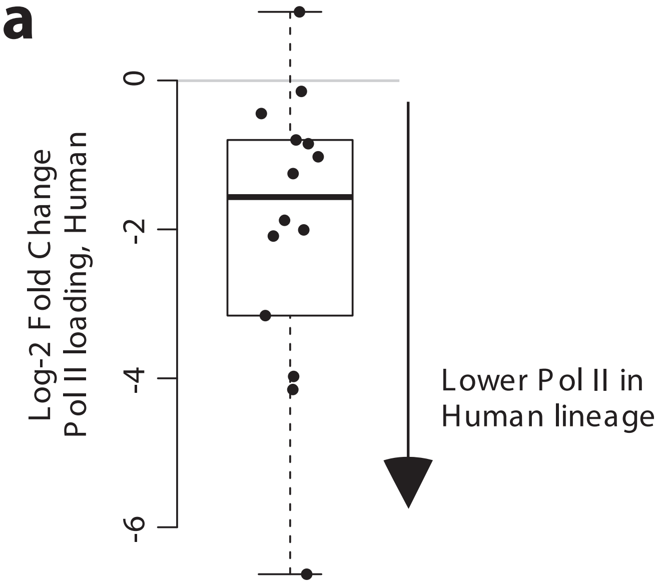

Supplementary Figure 10. Decrease Pol II Loading on *ANTXR2* CREs. a) Fourteen CREs based on Danko et al. in the *ANTXR2* locus show a bias for decreased Pol II loading in humans compared with non-human primates (chimpanzee and rhesus macaque). The Y axis denotes the log-2 fold-change in human. Values below 0 indicate decreased expression. The bold center line denotes the median value (50th percentile) and the boxes contain the 25th to 75th percentile of the data. The whiskers mark 1.5 times the interquartile range (IQR). Source data are provided as a Source Data file.

a

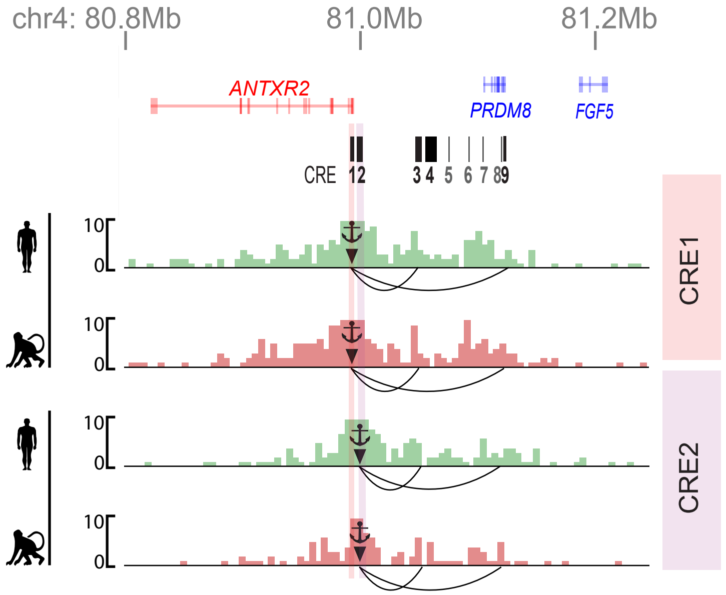

Supplementary Figure 11. Virtual 4C-seq of proximal cis-regulatory elements (CREs). a) Virtual 4C-seq plots of CRE1 and CRE2 show contact with the upstream CREs that had higher activity in non-human primates. Virtual 4C-seq signal calculated based on Hi-C contacts for CREs tested in the luciferase assay. CREs are colored. The anchor symbol at each CRE represents the bait region that all contacts are derived from. The arrow at the end of the arc denotes the *ANTXR2* promoter.

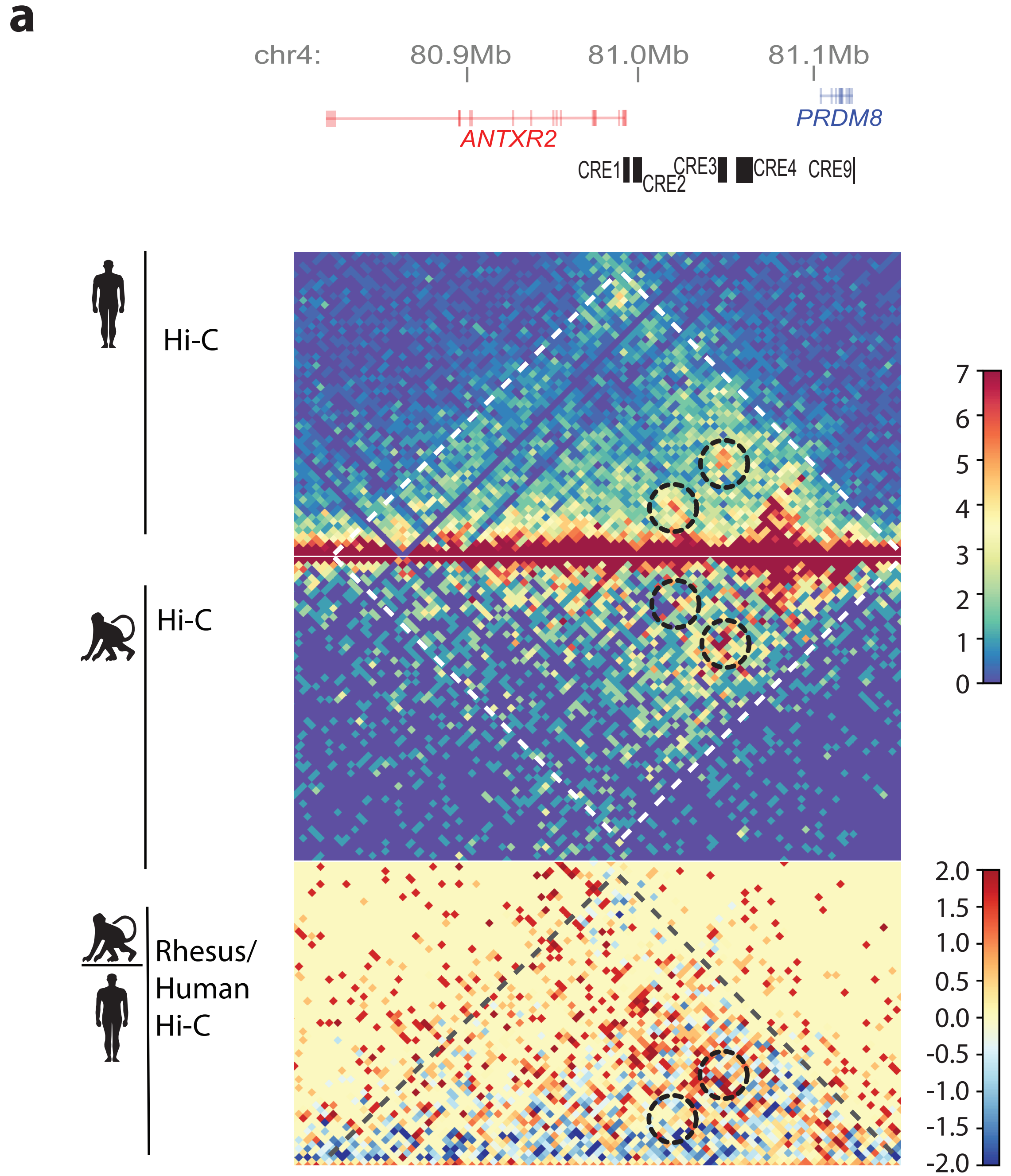

Supplementary Figure 12. Comparison of human and rhesus macaque Hi-C and Micro-C. a) Rhesus macaque Hi-C/Micro-C signal divided by human Hi-C/Micro-C shows an increase in contacts within the TAD containing *ANTXR2*. TADs are marked with white dotted lines (grey for human/rhesus macaque). Focal contacts with the *ANTXR2* promoter are circled with black dotted lines. Heatmaps represent normalized contacts.

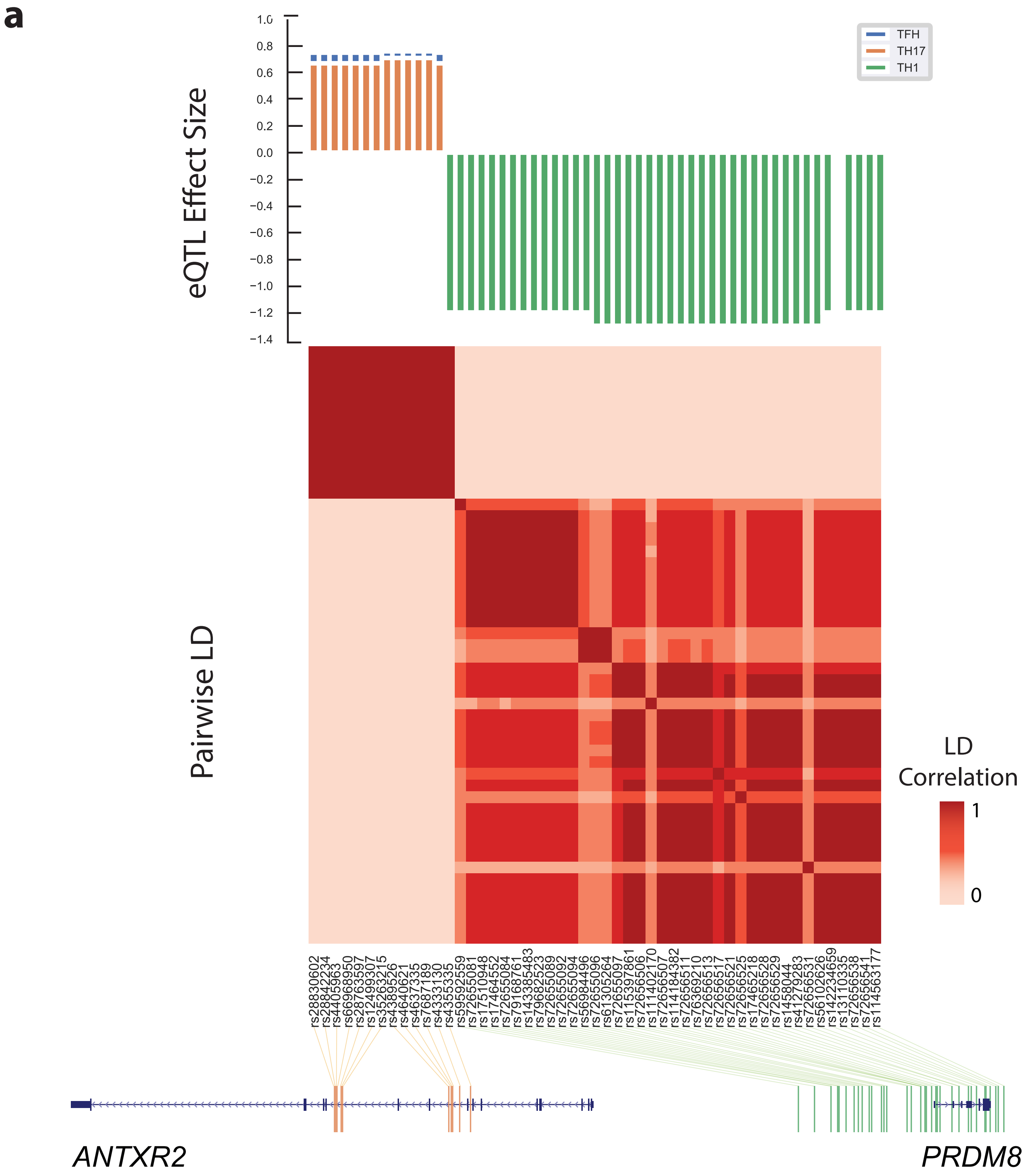

Supplementary Figure 13. *ANTXR2* eQTLs in humans. a) Top: eQTL (expression quantitative trait loci) effect size (calculated from the DICE database) in different T-cell populations (T folicular helper cells (TFH, blue), T helper cells 17 (TH17, orange), and T helper cells type 1 (TH1, green)). Bottom: Pairwise linkagedisequilibrium (LD) correlation heatmap between single nucleotide polymorphisms (SNPs) around the *ANTXR2* locus. Expression QTLs for *ANTXR2* fall into two main regions: within the gene and upstream of *ANTXR2* around *PRDM8*. Genic eQTLs are in LD and have a positive effect on *ANTXR2* expression. Upstream eQTLs fall within two blocks of LD and have a negative effect on ANTXR2 expression.

a

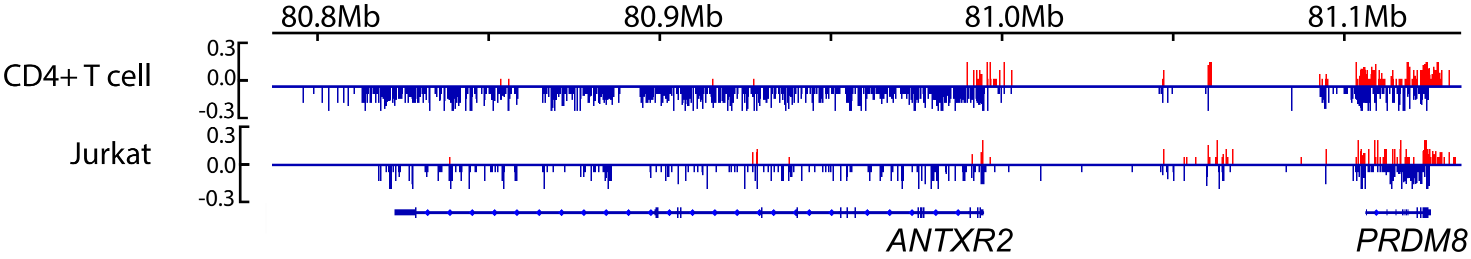

Supplementary Figure 14. Jurkat vs. CD4 PRO-seq. a) PRO-seq from Jurkat and human CD4+ T-cells shows a similar regulatory landscape around *ANTXR2*.

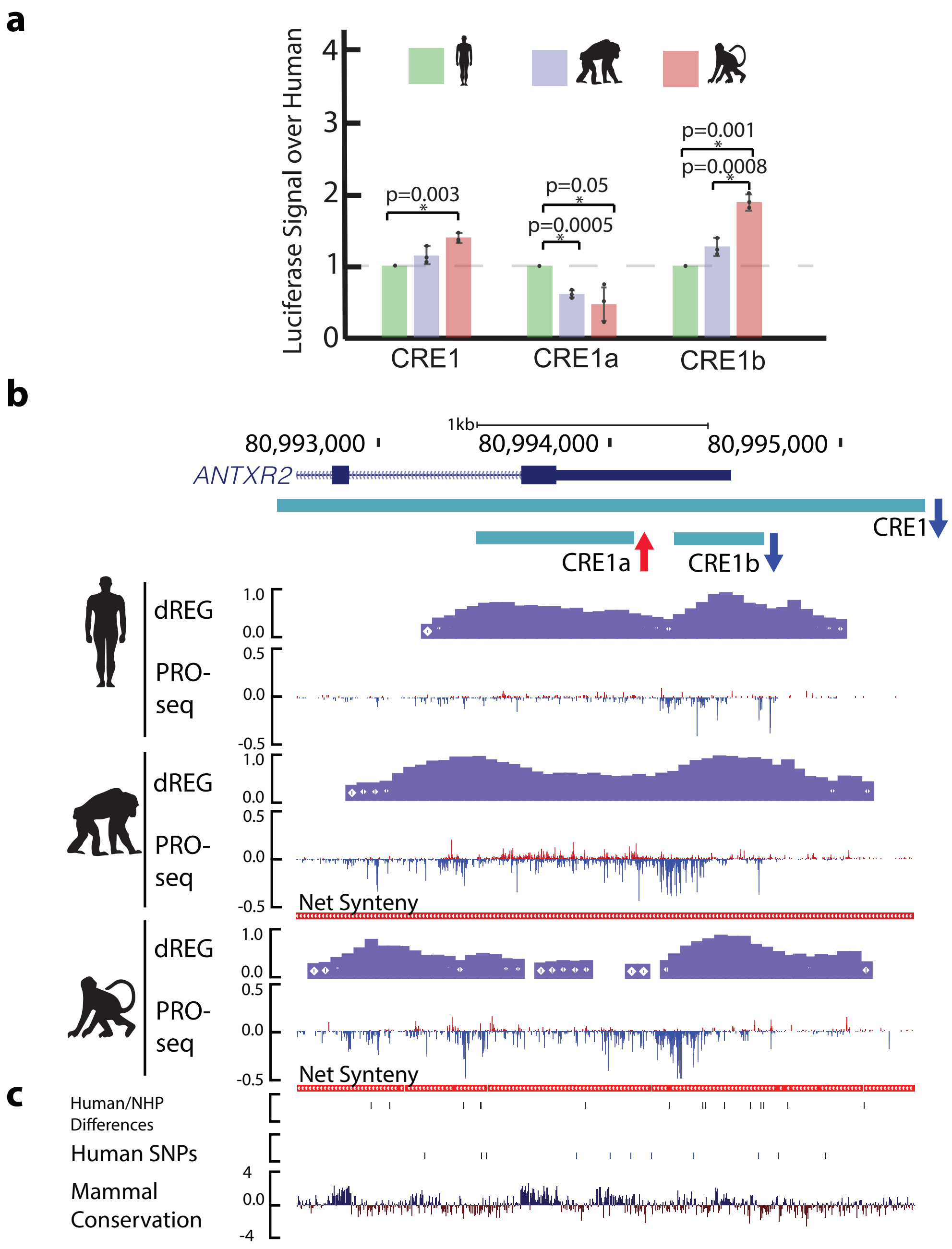

Supplementary Figure 15. Complex promoter of *ANTXR2*. a) Luciferase assay performed in Jurkat cells to test the activity of regulatory elements in human, chimpanzee, and rhesus macaque shows a human-specific decrease in the activity of cis-regulatory element 1 (CRE1). Sub-CREs 1a and 1b show a human-specific increase and decrease in activity, respectively. Data are presented as mean values +/- SE. One-sided t-tested used and significant p values are shown in the figure. n=3 independent transfections in each species. b) PRO-seq and dREG signal from human, chimpanzee, and rhesus macaque at CRE1 in the *ANTXR2* promoter. Net synteny tracks show the position of regions that have one-to-one orthologs in the chimpanzee and rhesus macaque genomes. c) Human-specific changes, human common single nucleotide polymorphisms (SNPs), and PhyloP conservation of the *ANTXR2* promoter. Source data are provided as a Source Data file.

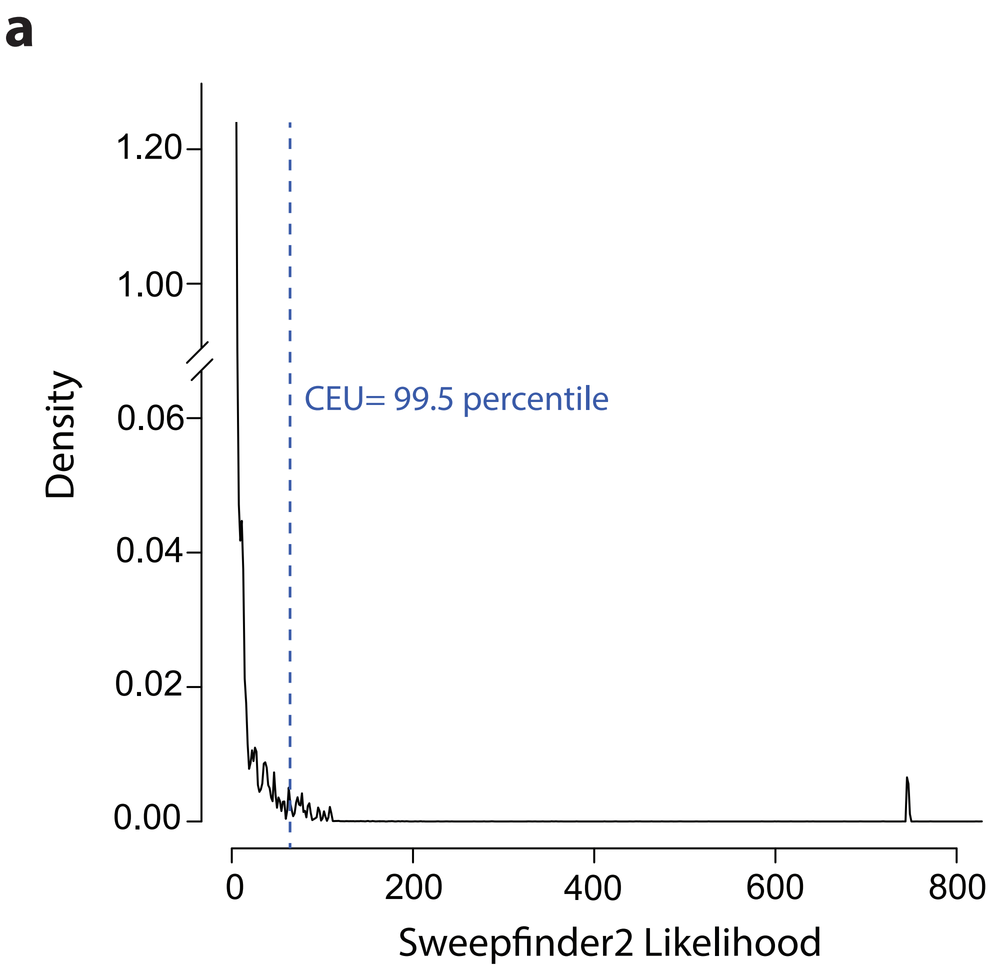

Supplementary Figure 16. CLR (composite-likelihood-ratio) percentile in CEU. a) The predicted selective sweep upstream of *ANTXR2* falls within the 99.5th percentile for all Sweepfinder2 likelihoods genome-wide.

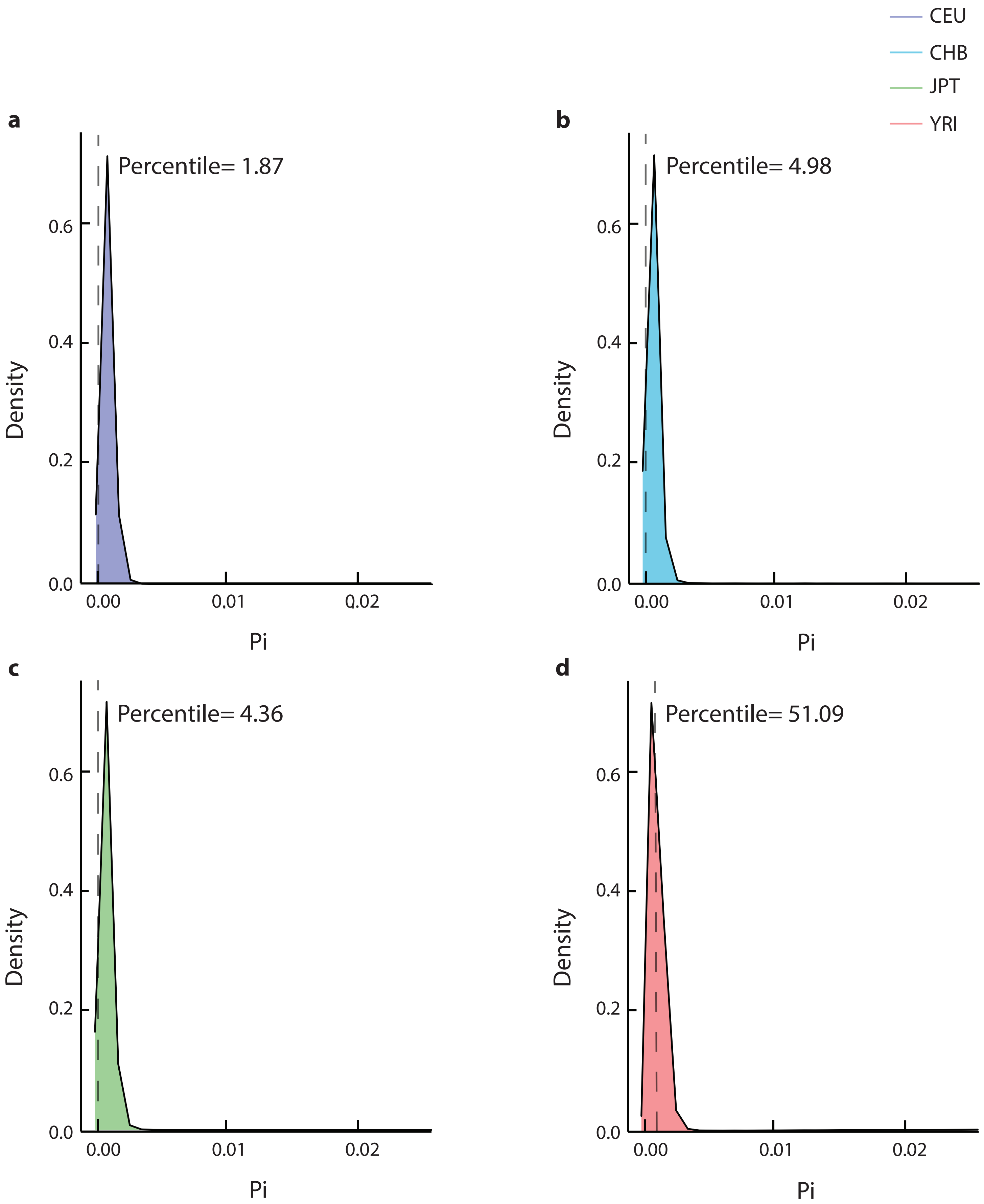

Supplementary Figure 17. Nucleotide diversity percentiles. a) Genome-wide values for Pi nucleotide diversity in CEU (Northern and Western Europe). The 5kb selective sweep falls in the 1.87th percentile. b) Genome-wide values for Pi nucleotide diversity in CHB (China). The 5kb selective sweep falls in the 4.98th percentile. c) Genome-wide values for Pi nucleotide diversity in JPT (Japan). The 5kb selective sweep falls in the 4.36th percentile. d) Genome-wide values for Pi nucleotide diversity in YRI (Yoruba). The 5kb selective sweep falls in the 51.09th percentile.

a

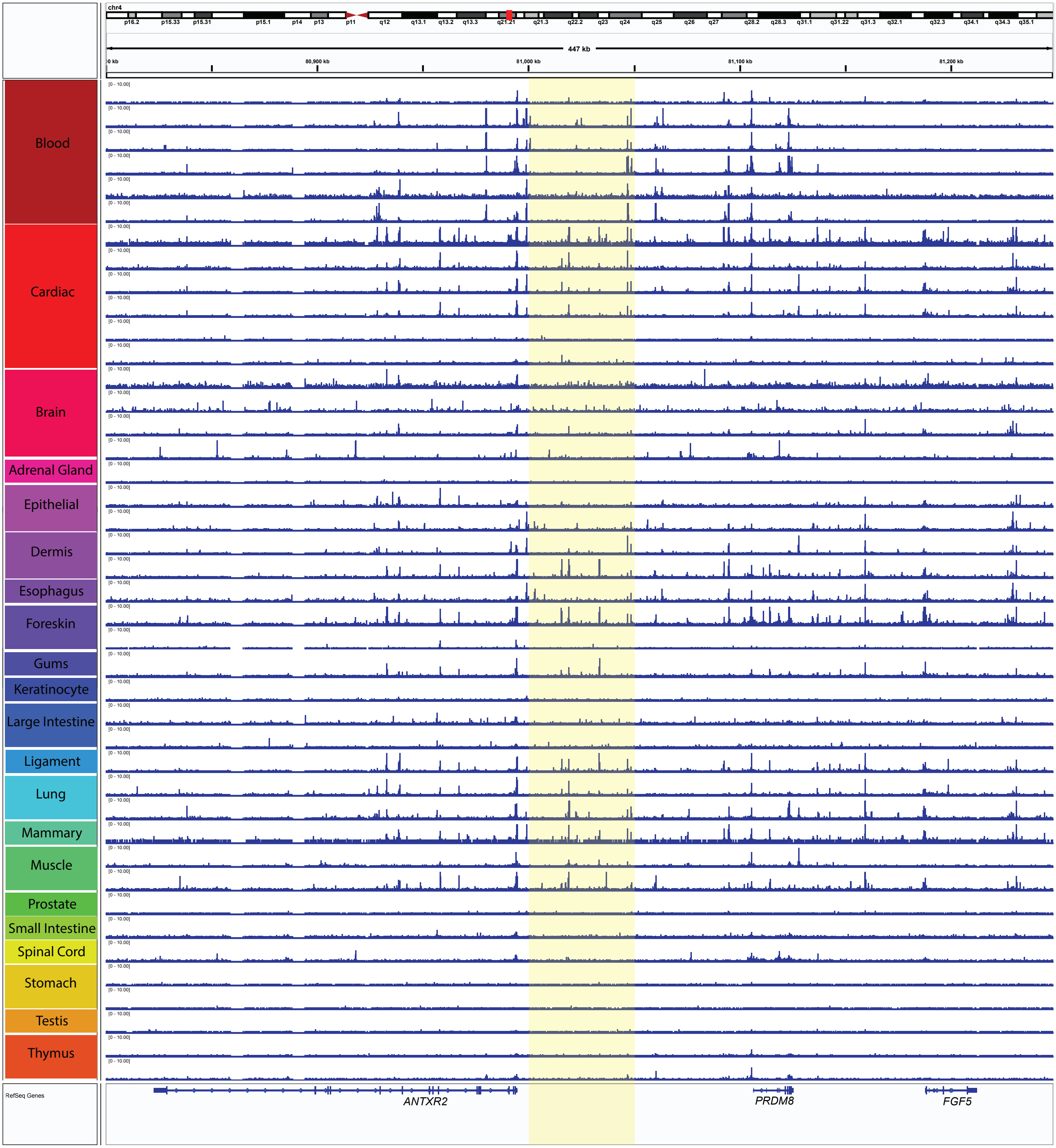

Supplementary Figure 18. DNase-I-seq profiles across diverse ENCODE tissues at *ANTXR2*. a) Patterns in DNase-I-seq data reveal differential regulatory landscapes between tissues at the *ANTXR2* locus. The 5kb selective sweep region is highlighted in yellow.

a

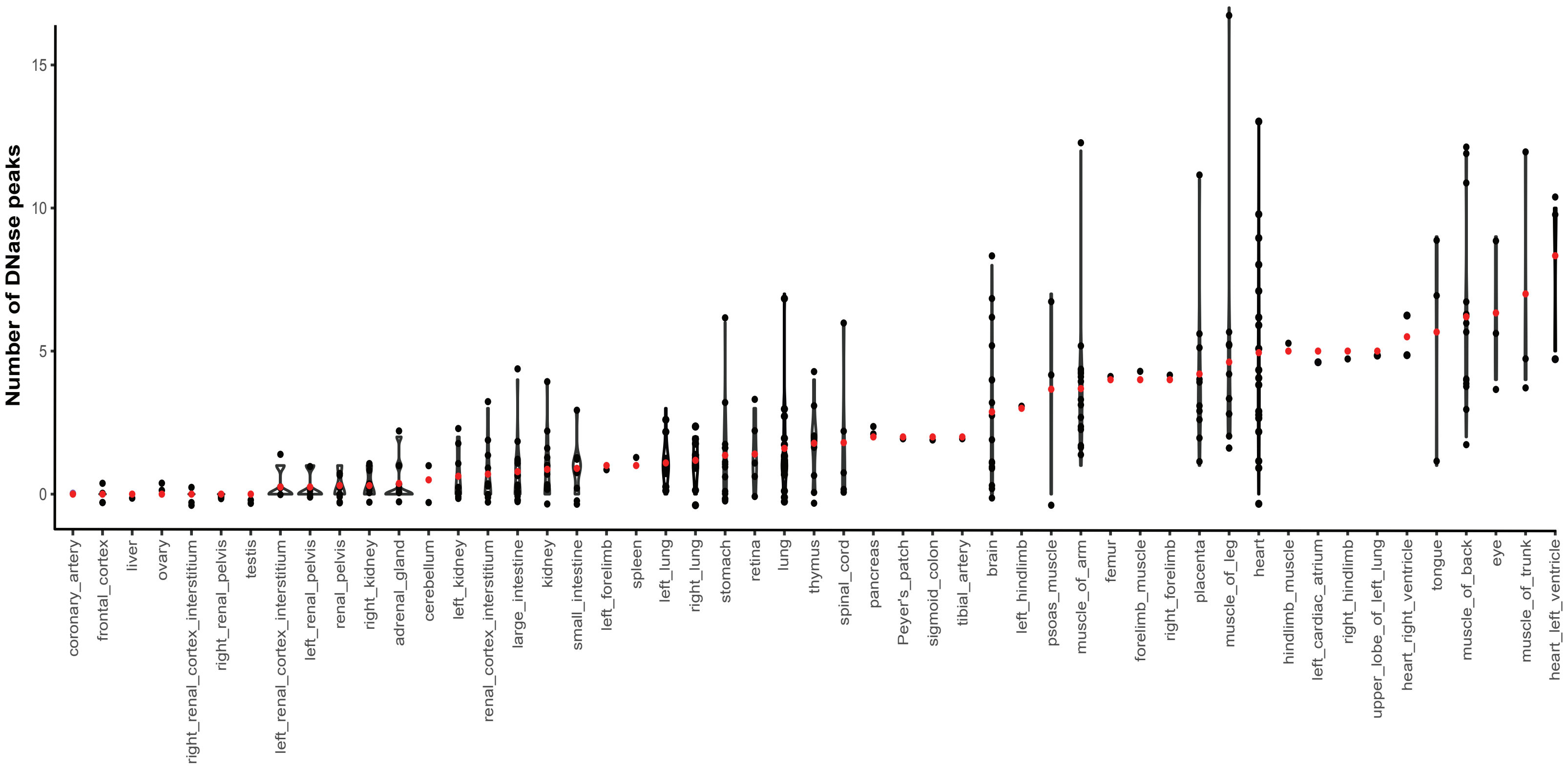

Supplementary Figure 19. Number of DNase-I hypersensitive sites within the composite-likelihood-ratio (CLR) interval across tissues. a) Number of DNase-I peaks in ENCODE tissue categories in the 5kb window of elevated CLR. Source data are provided as a Source Data file.

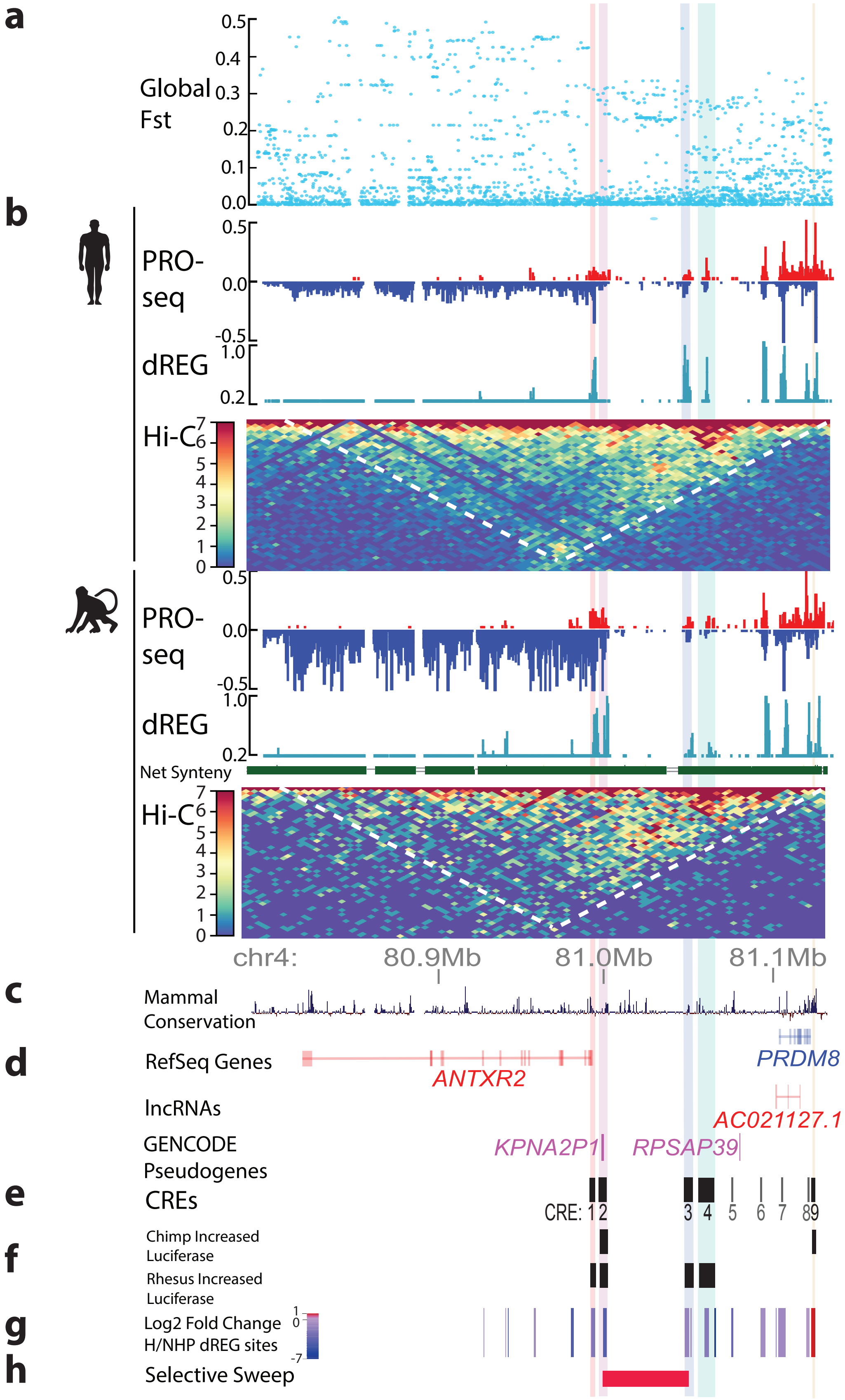

Supplementary Figure 20. Summary of findings at the *ANTXR2* locus. a) Global Fst (fixation index) values for all single nucleotide polymorphisms (SNPs) around the *ANTXR2* locus. b) Genome browser shot of CD4+ T-cell PRO-seq (normalized by reads per million, plus strand in red, minus strand in blue) in human and rhesus macaque. Regulatory elements predicted by dREG are shown in teal. c) 100-way conservation in mammals. d) RefSeq genes, lncRNA, and GENCODE Version 37 Pseudogenes. e) Cis-regulatory elements (CREs) tested in this study (CRE1-9) are shown below the data tracks. CREs with lower activity in human are shaded. f) CREs with lower activity in human than in chimpanzee or rhesus macaque as determined by luciferase. g) Log2 fold change of PRO-seq expression in humans compared to non-human primates for regulatory elements defined by Danko et al. h) Region of increased CLR in the CEU population (Northern and Western Europe).

	Human hg19	Rhesus rheMac10
Sequenced read pairs	1537590113	962448103
Normal paired	878369068	733240864
Chimeric paired	436270983	135018624
Chimeric ambiguous	164587895	59713541
Unmapped	58362167	34475074
Alignable (normal+chimeric)	1314640051	868259488
Hi-C contacts	741551222	351837113
Inter-chromosomal	159541332	77932242
Intra-chromosomal	582009890	273904871
Short range (<20Kb)	342099230	175012244
Long range (>20Kb)	239059227	98875560

Supplementary Table 1. Hi-C and Micro-C mapping statistics.
For each species, individuals were combined using the Juicer pipeline. Human Hi-C contacts totalled 741,551,222 and Rhesus Macaque contacts totaled 351,837,113.

Primer name	Sequence
CRE_1_HCR_for	TCGAGCTCTTTTGATCATCCAAGCCTGTT
CRE_1_HCR_rev	TCAGCCGTCAGCCAGACTGTCAGCTTCA
CRE_1.1_H_for	GGCTCGAGCTTCAATGCCACGAACATAGGG
CRE_1.1_R_for	GGCTCGAGCTTCAATGCCACGAACATGGGG
CRE_1.1_C_for	GGCTCGAGCTTCAATGTCACGAACATGGGG
CRE_1.1_HC_rev	ACACGCGTGAGCTTAGGGGACTGCGAGC
CRE_1.1_R_rev	ACACGCGTGAGCTGAAGAGACTGCGAGC
CRE_1.2_HC_for	CAACGCGTCAGCTCGCGAAAGGAGTTC
CRE_1.2_R_for	CAACGCGTCAGCTCGCGAGATGAGTTC
CRE_1.2_HR_rev	GAGGTACCCGCTGGAGGCTCGGC
CRE_1.2_C_rev	CAGGTACCCGCTGGAGGCTCGGC
CRE_2_HC_for	TCACGCGTGAGAACTGAAACAGGCTGACA
CRE_2_R_for	TCACGCGTCAGAACTGAAACAGGCTGACA
CRE_2_HCR_rev	AGCTCGAGTCTGCCTTCCAGTGTTGACTC
CRE_3_HC_for	GAGAGCTCGCCATAAGTCTCCCATCCAA
CRE_3_R_for	TAGAGCTCACCATAAGTCTCCCATCCAA
CRE_3_HC_rev	TTAGCCGTTGCCAGAAAAGTTCCTGCTT
CRE_3_R_rev	TTAGCCGTTGCCAGAAAAGGTCCTGCTT
CRE_4_HCR_for	GAACGCGTCAGGCCTGCAAGCTTCCTAT
CRE_4_HC_rev	CTCTCGAGACTGCCTCCCCCTTAAGTCT
CRE_4_R_rev	CTCTCGAGACTGCCTCCCCCTTAACTCT
CRE_5_HC_for	CTACGCGTCAGTGGTGAGTAGTACGAGCC
CRE_5_R_for	CTACGCGTTAGTGGTGAGTAGTATGAGCC
CRE_5_H_rev	TAGGTACCCTAGACACCAGGCAGTCACG
CRE_5_C_rev	TAGGTACCCTAGACAACATGCAGTCACG
CRE_5_R_rev	TAGGTACCGTAGACAACATGCAGTCACG
CRE_6_H_for	TCACGGCTGTGAGGCACAGTTCTGGGTA
CRE_6_C_for	TCACGGCTGTGAGGCACAGTTCTGGGTG
CRE_6_R_for	TCACGGCTGGGAGGCACAGTTCTGAGTG
CRE_6_HC_rev	CGAGAGAGTGAGGGACACAGAAACCAGC
CRE_6_R_rev	TGAGAGAGTGAGGGACACAAGAGCTAGC
CRE_7_HC_for	CCACGCGTCTGGAAAGCGGTAATGCCCA
CRE_7_R_for	CCACGCGTCTGGAAAGCGGTAATGCCCT
CRE_7_HCR_rev	TGGGTACCTAGAGAGGACATCGGACGGG
CRE_8_HC_for	AGACGCGTAGATAGGTGAGGATAAAGCGAGG
CRE_8_R_for	AGACGCGTAGATAGGTGAAGATAAAGCAAGG
CRE_8_HCR_rev	CGGGTACCAGAGGCGGCCGTTCTCTTC
CRE_9_HCR_for	GGACGCGTCGGGGCCCTTGCAGC
CRE_9_HCR_rev	CTGGTACCTGGGGAAAGTGAAAATGCGTG
ANTXR2_CRISPRa_for	acacCGTGACACACGCAGCCCAGACCG
ANTXR2_CRISPRa_rev	aaaaCGGTCTGGGCTGCGTGTGTCACG
ANTXR2_qRT-PCR_for	CCCACCAGGCTAAGCAAATA
ANTXR2_qRT-PCR_rev	TCCAGTCAGAAACTTCCCATAAG

Supplementary Table 2. Sequences of primers used in this study.
Primers named CRE_#_species (H=human, C=chimp, R=rhesus)_direction were used to amplify regions of interest for the luciferase assay. One primer pair was used for all species when there were no SNPs within the primer. When SNPs were present, species-specific primers were used to amplify the same region. Primers ANTXR2_CRISPRa_for and ANTXR2_ CRISPRa _rev were used generating guide RNAs for CRISPRa experiments. Primers ANTXR2_qRT-PCR_for and ANTXR2_qRT-PCR_rev were used for measuring *ANTXR2* expression after CRISPRa experiments.
