# Supplementary material for: Multiple stages of evolutionary change in anthrax toxin receptor expression in humans
Source: Nat Commun. 2021 Nov 15;12:6590. doi: 10.1038/s41467-021-26854-z (PMC8592990; doi:10.1038/s41467-021-26854-z)
Supplement: Supplementary file 3 — Reporting Summary [file 41467_2021_26854_MOESM3_ESM.pdf]

nature research

Corresponding author(s): Danko and Lewis

Last updated by author(s): Aug 3, 2021

Reporting Summary

Nature Research wishes to improve the reproducibility of the work that we publish. This form provides structure for consistency and transparency in reporting. For further information on Nature Research policies, see our Editorial Policies and the Editorial Policy Checklist.

Statistics

For all statistical analyses, confirm that the following items are present in the figure legend, table legend, main text, or Methods section.

n/a	Confirmed	
☐	☒	The exact sample size (*n*) for each experimental group/condition, given as a discrete number and unit of measurement
☐	☒	A statement on whether measurements were taken from distinct samples or whether the same sample was measured repeatedly
☐	☒	The statistical test(s) used AND whether they are one- or two-sided<br>*Only common tests should be described solely by name; describe more complex techniques in the Methods section.*
☐	☒	A description of all covariates tested
☐	☒	A description of any assumptions or corrections, such as tests of normality and adjustment for multiple comparisons
☐	☒	A full description of the statistical parameters including central tendency (e.g. means) or other basic estimates (e.g. regression coefficient) AND variation (e.g. standard deviation) or associated estimates of uncertainty (e.g. confidence intervals)
☐	☒	For null hypothesis testing, the test statistic (e.g. F, t, r) with confidence intervals, effect sizes, degrees of freedom and P value noted<br>*Give P values as exact values whenever suitable.*
☐	☒	For Bayesian analysis, information on the choice of priors and Markov chain Monte Carlo settings
☐	☒	For hierarchical and complex designs, identification of the appropriate level for tests and full reporting of outcomes
☒	☐	Estimates of effect sizes (e.g. Cohen's *d*, Pearson's *r*), indicating how they were calculated

Our web collection on statistics for biologists contains articles on many of the points above.

Software and code

Policy information about availability of computer code

Data collection	No software was used during data collection.
Data analysis	Hi-C data was analyzed using the Juicer package version 1.6. PRO-seq data was analyzed using BWA (version 0.7.17), Kent source software tools (version 1.04.00), BEDTools (version 2.29.2), dREG (version 2), and DESeq2 (version 1.26.0). CrossMap (version 0.4.2) was used to convert between genome coordinates. RNA-seq data was quantified using Salmon (version 0.14.1) or STAR (version 2.7.2) and DESeq2 (version 1.26.0). Sweepfinder2 (version 1.0) was used to detect selective sweeps. VCFtools (version 0.1.16) was used to calculate Fst values.<br><br>All custom code used in analysis is available at: https://github.com/Danko-Lab/ANTXR2

For manuscripts utilizing custom algorithms or software that are central to the research but not yet described in published literature, software must be made available to editors and reviewers. We strongly encourage code deposition in a community repository (e.g. GitHub). See the Nature Research guidelines for submitting code & software for further information.

Data

Policy information about availability of data

All manuscripts must include a data availability statement. This statement should provide the following information, where applicable:

- Accession codes, unique identifiers, or web links for publicly available datasets
- A list of figures that have associated raw data
- A description of any restrictions on data availability

Data generated in this study:
The rhesus macaque Hi-C, Micro-C, and RNA-seq and the baboon RNA-seq data generated in this study have been deposited in the GEO database under accession code GSE156161 [https://www.ncbi.nlm.nih.gov/geo/query/acc.cgi?acc=GSE156161]. The processed data is also available under accession code GSE156161. The

human Hi-C, Micro-C, and RNA-seq data generated in this study have been deposited in dbGaP under project number phs002146.v1.p1 [https://www.ncbi.nlm.nih.gov/projects/gap/cgi-bin/study.cgi?study_id=phs002146.v1.p1], access can be obtained by requesting access to dbGaP. The CRISPRa, luciferase, and other functional assay data generated in this study are provided in the Source Data file and at https://github.com/Danko-Lab/ANTXR2. Each figure's data is in it's own excel sheet.

Publicly available data:
The PRO-seq data used in this study are available in the GEO database under accession code GSE85337 [https://www.ncbi.nlm.nih.gov/geo/query/acc.cgi?acc=GSE85337]. The DICE human immune cell RNA-seq data used in this study are available in the dbGaP database under project number phs001703.v1.p1 [https://www.ncbi.nlm.nih.gov/projects/gap/cgi-bin/study.cgi?study_id=phs001703.v1.p1], access can be obtained by requesting access to dbGaP. The rhesus macaque immune cell RNA-seq data used in this study are available in the GEO database under accession code GSE83302 [https://www.ncbi.nlm.nih.gov/geo/query/acc.cgi?acc=GSE83302]. The cross-species expression microarray data used in this study are available in the GEO database under accession code GSE11560 [https://www.ncbi.nlm.nih.gov/geo/query/acc.cgi?acc=GSE11560]. The Bakiga and Batwa population RNA-seq data used in this study are available in the GEO database under accession code GSE120502 [https://www.ncbi.nlm.nih.gov/geo/query/acc.cgi?acc=GSE120502]. Rhesus macaque PBMC RNA-seq data used in this study are available in the BioProject database under accession code PRJNA246101 [https://www.ncbi.nlm.nih.gov/bioproject/PRJNA246101]. Human DNase-1-seq data used in this study are available in the ENCODE database [https://www.encodeproject.org/], individual accession codes can be fund in the Source Data file. The human tissue RNA-seq data used in this study are available from GTEx (https://gtexportal.org/home/datasets).
The CD4+ T-cell H3K27ac data used in this study are available in the GEO database under accession code GSE40668 (https://www.ncbi.nlm.nih.gov/geo/query/acc.cgi?acc=GSE400668). The CD4+ primary T-cell DNase-I-seq data used in this study are available in the GEO database under accession code GSM736592 (https://www.ncbi.nlm.nih.gov/geo/query/acc.cgi?acc=GSM736592). The Jurkat PRO-seq data used in this study are available in the GEO database under accession code GSE66031 (https://www.ncbi.nlm.nih.gov/geo/query/acc.cgi?acc=GSE66031). The K562 PRO-seq data used in this study are available in the GEO database under accession code GSE60456 (https://www.ncbi.nlm.nih.gov/geo/query/acc.cgi?acc=GSE60456).

Field-specific reporting

Please select the one below that is the best fit for your research. If you are not sure, read the appropriate sections before making your selection.

☒ Life sciences ☐ Behavioural & social sciences ☐ Ecological, evolutionary & environmental sciences

For a reference copy of the document with all sections, see nature.com/documents/nr-reporting-summary-flat.pdf

Life sciences study design

All studies must disclose on these points even when the disclosure is negative.

Sample size	A statistical power test was not conducted. The two to three individuals were selected for each species to account for within-species variation, and provide enough statistical power to identify medium-sized differences between species. Two samples were collected for genomic experiments because this was the number available from primate centers.
Data exclusions	No data were excluded from the analysis.
Replication	Each experiment was replicated according to the number of times reported in the methods section. A minimum of two biological replicatess were performed for each species. All attempts at replication were successful.
Randomization	Samples were allocated to groups based on the species from which each sample was derived.
Blinding	Blinding is not applicable to this study. Sample processing required knowledge of which sample belonged to each species to allow the appropriate reference genome for use in analysis.

Reporting for specific materials, systems and methods

We require information from authors about some types of materials, experimental systems and methods used in many studies. Here, indicate whether each material, system or method listed is relevant to your study. If you are not sure if a list item applies to your research, read the appropriate section before selecting a response.

Materials & experimental systems

n/a	Involved in the study	
☐	☒	Antibodies
☐	☒	Eukaryotic cell lines
☒	☐	Palaeontology and archaeology
☐	☒	Animals and other organisms
☐	☒	Human research participants
☒	☐	Clinical data
☒	☐	Dual use research of concern

Methods

n/a	Involved in the study	
☒	☐	ChIP-seq
☒	☐	Flow cytometry
☒	☐	MRI-based neuroimaging

Antibodies

Antibodies used	Cell separation was performed using species-specific antibodies pre-conjugated to microbeads by the manufacturer (Miltenyi Biotech). Miltenyi item numbers used are Human= 130-045-101 and Rhesus= 130-091-102
Validation	Results were validated by flow cytometry using a second CD4 antibody as reported in Danko et al. 2018.

Eukaryotic cell lines

Policy information about cell lines

Cell line source(s)	K562 and Jurkat cell lines were originally purchased from ATCC. Phoenix-AMPHO were a gift from Dr. Scott Coonrod's lab.
Authentication	Cell lines were not authenticated.
Mycoplasma contamination	All cell lines tested negative for mycoplasma contamination.
Commonly misidentified lines (See ICLAC register)	No commonly misidentified cell lines were used in the study

Animals and other organisms

Policy information about studies involving animals; ARRIVE guidelines recommended for reporting animal research

Laboratory animals	Blood samples from Indian rhesus macaques (n=2, 1 male and 1 female, age > 2 years) and Anubis baboons (n=4, 2 males and 2 females, age > 2 years) were used in this study. Data from chimpanzees was from Danko et al. 2018 (PMID: 29379187).
Wild animals	This study did not involve wild animals.
Field-collected samples	This study did not involve samples collected from the field.
Ethics oversight	This work was approved by the Cornell University IACUC.

Note that full information on the approval of the study protocol must also be provided in the manuscript.

Human research participants

Policy information about studies involving human research participants

Population characteristics	The human subjects included in this study were healthy adults from Cornell University (n=4, 2 males and 2 females). Participants filled out a health survey prior to participation in the study. All participants were > 25 years old but exact ages were not recorded. Demographic parameters represent those in Ithaca NY from which samples were drawn.
Recruitment	Participants were recruited through emails to the Life Sciences departments at Cornell University. While this method allowed for self selection bias, the demographic of volunteers matched that of the Ithaca NY area.
Ethics oversight	This work was approved by the Cornell University IRB.

Note that full information on the approval of the study protocol must also be provided in the manuscript.
